# Diabetes-induced male infertility: potential mechanisms and treatment options

**DOI:** 10.1186/s10020-023-00771-x

**Published:** 2024-01-15

**Authors:** Runchun Huang, Jiawang Chen, Buyu Guo, Chenjun Jiang, Weiming Sun

**Affiliations:** 1 The First Clinical Medical College, Lanzhou University, Lanzhou, People’s Republic of China 730000; 2 Department of Endocrinology, The First Hospital of Lanzhou University, Lanzhou, 730000 Gansu People’s Republic of China

**Keywords:** Diabetes, Male infertility, Hyperglycemia, Oxidative stress, Chronic inflammation, Endocrine disorders, Sperm

## Abstract

Male infertility is a physiological phenomenon in which a man is unable to impregnate a fertile woman during a 12-month period of continuous, unprotected sexual intercourse. A growing body of clinical and epidemiological evidence indicates that the increasing incidence of male reproductive problems, especially infertility, shows a very similar trend to the incidence of diabetes within the same age range. In addition, a large number of previous in vivo and in vitro experiments have also suggested that the complex pathophysiological changes caused by diabetes may induce male infertility in multiple aspects, including hypothalamic-pituitary–gonadal axis dysfunction, spermatogenesis and maturation disorders, testicular interstitial cell damage erectile dysfunction. Based on the above related mechanisms, a large number of studies have focused on the potential therapeutic association between diabetes progression and infertility in patients with diabetes and infertility, providing important clues for the treatment of this population. In this paper, we summarized the research results of the effects of diabetes on male reproductive function in recent 5 years, elaborated the potential pathophysiological mechanisms of male infertility induced by diabetes, and reviewed and prospected the therapeutic measures.

## Introduction

Diabetes is a chronic metabolic disease characterized by chronic hyperglycemia secondary to absolute/relative insulin deficiency and/or insulin resistance in the context of islet beta cell dysfunction, often accompanied by metabolic syndrome (Classification and Diagnosis of Diabetes 2022). It may be diagnosed based on plasma glucose criteria, either the fasting plasma glucose (FPG) value or the 2-h plasma glucose (2-h PG) value during a 75-g oral glucose tolerance test (OGTT) or A1C criteria (ElSayed et al. 2023). In recent decades, the prevalence of diabetes has shown a rapid rise and has become one of the most prominent public health threats in modern society (Zheng et al. 2018). According to 2021 statistics, 573 million adults (20–79 years old) worldwide have diabetes, and the number of people with diabetes is expected to rise to 643 million by 2030.In 2045, it will rise to 783 million (Saeedi et al. 2019).

The occurrence and development of diabetes involves a variety of pathophysiological mechanisms, including hyperglycemia, dyslipidemia, hypertension, oxidative stress, chronic inflammation, mitochondrial dysfunction, and endoplasmic reticulum(ER) stress. These mechanisms interact with each other, leading to large vascular disease, microangiopathy and neuropathy (Defeudis et al. 2022), thus causing multiple organ and tissue damage, dysfunction, and even failure in the whole body.Epidemiological studies have found that diabetic men have a higher risk of infertility compared to non-diabetic men (Bener et al. 2009; Sexton and Jarow 1997). At the same time, evidence from diabetic patients and animal models also demonstrates that diabetes has a significant impact on the reproductive system, including dysfunction of the hypothalamic-pituitary–gonadal (HPG) axis, decreased testosterone synthesis and secretion, testicular failure, spermatogenesis disorders, erectile dysfunction (ED), and Ejaculatory disorder and other factors (Sexton and Jarow 1997; Ding et al. 2015; Rodrigues et al. 1995). These results result from both direct and indirect effects of diabetes. Diabetic neuropathy (especially autonomic and peripheral neuropathy) appears to be the most commonly involved indirect mechanism, widely mediating the occurrence of ED (Bleustein et al. 2002), ejaculatory complications(Shamloul and Ghanem 2013; Ledda 2000).

With the increasing incidence of diabetes, especially in recent years, the incidence of diabetes at a younger age trend (Al-Saeed et al. 2016), the problems of male infertility of childbearing age may become more common. However, a comprehensive overview of the pathophysiology, consequences and treatment of diabetes-induced male infertility is currently lacking. The purpose of this review is to elucidate the complex complications of diabetes and the potential mechanisms leading to male infertility, and to summarize the known therapeutic measures, so as to provide new ideas for exploring more effective ways to prevent and treat diabetes-induced male infertility.

## Complications of diabetes: complex pathophysiological changes

**Fig. 1:**
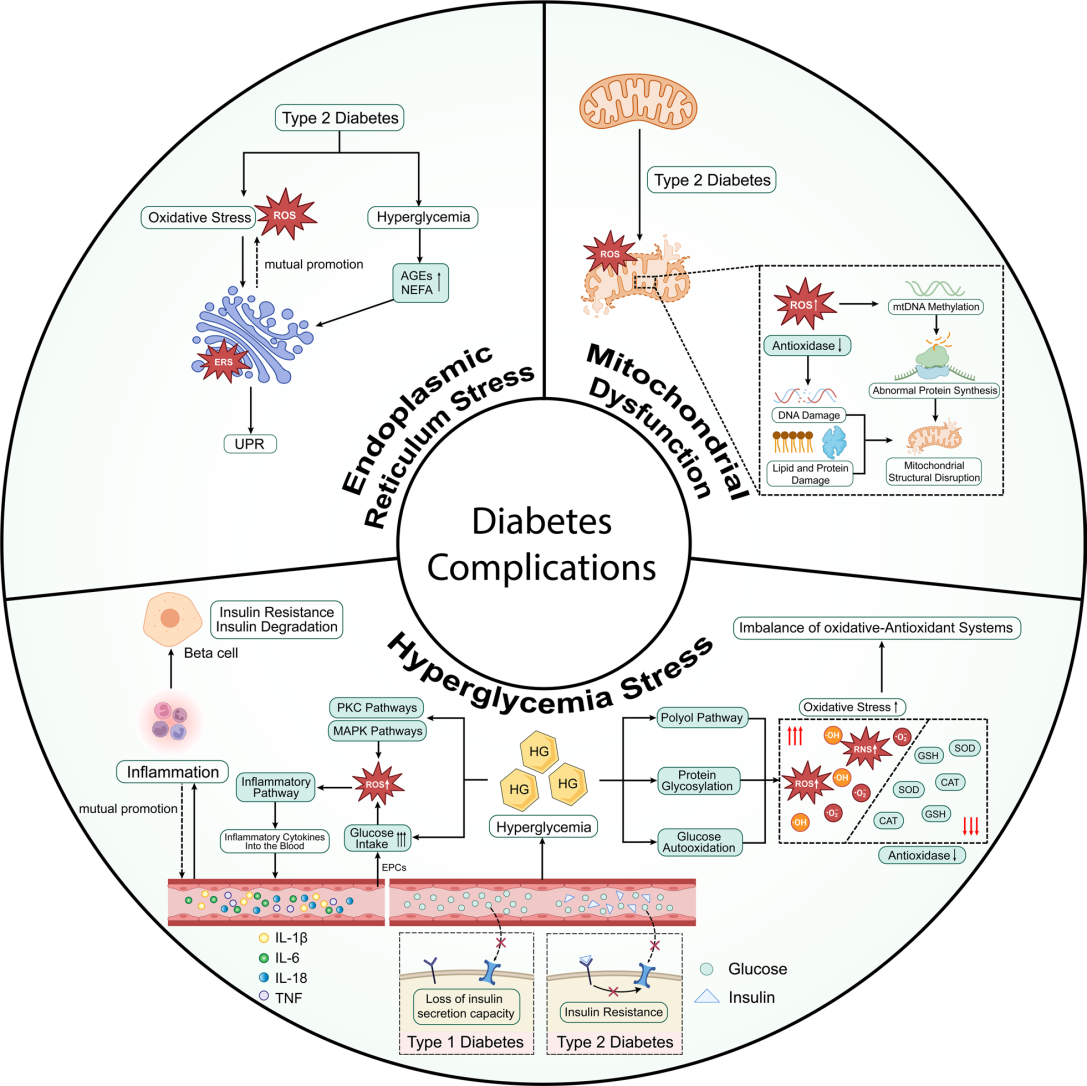
Diabetic comorbidities: Complex pathophysiological changes. AGEs: advanced glycation end products; NEFA: nonestesterified fatty acid; UPR: unfolded protein response; ROS: reactive oxygen species; RNS: reactive nitrogen species; HG: high glucose; TNF: tumor necrosis factor; PKC: Protein kinase C; MAPK: Mitogen-activated protein kinase; CAT: catalase; SOD: superoxide dismutase; GSH: glutathione

Diabetes, as a chronic metabolic disease, starts with hyperglycemia and induces complex pathophysiological changes in the body, including hyperglycemia stress, mitochondrial dysfunction and ER stress.These factors influence each other to form a complex network of cellular and molecular signals (Fig. 1).

### Hyperglycemia stress

The most important feature of diabetes is high blood glucose and it is caused by glucose metabolism disorders. In physiological conditions, the maintenance of normal glucose metabolism depends on the interaction of insulin, glucagon, islet amyloid, and incretin (Aronoff et al. 2004). Insulin is the only known hormone in the body that can lower blood glucose levels.

It is produced by β cells, which involves a maturation process in which insulin precursors are transformed into proinsulin through conformational modification, then into immature secretory vesicles and cleaved to C-peptide and insulin (Fu et al. 2013). The release of insulin is mainly triggered by the response of β cells to high glucose concentrations, other factors include amino acids, fatty acids and hormones (Boland et al. 2017).

In patients with type 1 diabetes (T1D), autoimmune destruction of islet β cells in the body leads to loss of insulin secretion ability, resulting in hyperglycemia (Maresch et al. 2019). Patients with type 2 diabetes (T2D) also develop β-cell dysfunction leading to varying degrees of insulin insufficiency or occur insulin resistance, which may be the result of complex interactions between the environment and different molecular pathways involved in cell biology, including a long-term high-glucose, high-fat diet, oxidative stress, chronic inflammation, and epigenetic disruption and imbalances (Maresch et al. 2019; Halban et al. 2014). A growing body of evidence highlights the importance of miRNA-mediated post-transcriptional regulation for a variety of physiological functions of β cells, including glucose metabolism and insulin synthesis and secretion (LaPierre and Stoffel 2017). This suggests that dysregulation of miRNA expression may directly impair β cell function, leading to the occurrence of hyperglycemia under diabetes conditions (Esguerra et al. 2018). For example, overexpression of miR-375 leads to impaired exocytosis in β cells, which reduces insulin secretion. However, downregulation of miR-375 expression also results in a decrease in β cell mass (Poy et al. 2009).

Studies have found that a state of high blood glucose can activate oxidative stress.

Oxidative stress refers to a state of imbalance between oxidation (i.e. free radical formation) and antioxidant defense in the body (Yaribeygi et al. 2020), tending to oxidation, resulting in inflammatory infiltration of neutrophils, increased protease secretion, and the production of a large number of oxidative intermediates. The so-called free radicals refer to the active derivatives of oxygen molecules such as reactive oxygen species (ROS) and nitrogen molecules such as reactive nitrogen (RNS) and peroxynitrite (Gutteridge and Halliwell 1992). Normal levels of free radicals are involved in many molecular pathways in the body, including intracellular signaling, immune defense, cell growth, autophagy, apoptosis, and senescence (Bókkon 2012; Brown et al. 2004). The antioxidant defense in the body mainly depends on various antioxidant enzymes in the cell, such as glutathione (GSH), superoxide dismutase (SOD), catalase (CAT), which can protect cells from damage caused by free radicals (Maritim et al. 2003). When the formation of free radicals exceeds the action of antioxidant enzymes, oxidative stress is induced. ROS production includes polyol pathway, protein glycosylation and glucose autoxidation (Nawale et al. 2006; Giacco and Brownlee 2010). It has been shown that these pathways are over-activated in diabetic hyperglycemia, leading to excessive production of ROS and causing tissue oxidative damage. Based on the analysis of relevant experimental and clinical studies, the main pathway of oxidative damage of pancreatic β cells and endothelial dysfunction is also the main source of ROS increase and oxidative stress in diabetes (Darenskaya et al. 2021).

Diabetes, especially T2D, is widely recognized as an inflammatory disease (Germolec et al. 2018). Hyperglycemia is manifested by a range of lesions, the most prominent of which is chronic inflammation (Tsalamandris et al. 2019). On the one hand, high blood glucose is involved in the production of inflammation throughout the body. The study reported that the levels of IL-1β (pro-inflammatory cytokine) was increased and the level of IL-10 (anti-inflammatory cytokine) was decreased in diabetic rats (Nna et al. 2020). In artificially induced hyperglycemia, the levels of IL-6, TNF and IL-18 in human plasma increased sharply, which may be related to the mechanism of oxidative stress: Under the condition of hyperglycemia, the uptake of glucose by endothelial cells increases, resulting in excessive production of ROS in mitochondria, which causes oxidative damage and activates the inflammatory signaling cascade in endothelial cells, releasing a large number of inflammatory cytokines into the blood (Esposito et al. 2002; Donath and Shoelson 2011). In addition, high glucose can also induce the transcription and activation of multiple monocyte pro-inflammatory cytokines and chemokine-related genes through key signaling pathways (oxidative stress, PKC pathway, MAPK pathway, etc.), which may ultimately increase the activation and adhesion of monocytes (Shanmugam et al. 2003), which is conducive to the spread of inflammatory response. On the other hand, inflammation promotes the development of diabetes. Various inflammatory cytokines produced during inflammation, such as TNF-α, IL-1β and IL-6, can affect the function of islet β cells, promote insulin resistance and insulin degradation through specific pathways (Aguirre et al. 2000; Kurauti et al. 2017).

### Mitochondrial dysfunction

As an important intracellular organelle, mitochondria are involved in a variety of cellular life activities, such as cell respiration and energy substrate metabolism, ROS production, apoptosis, and signal transduction involved in cell proliferation (Zhang et al. 2019). T2D leads to systemic mitochondrial dysfunction through complex mechanisms (Pinti et al. 2019), which in turn leads to energy substrate metabolism dysfunction, ROS overproduction and cell proliferation inhibition, etc., resulting in dysfunction of various organs in the whole body, such as liver cell metabolism disorders, decreased cardiac output, and damaged skeletal muscle contractions (Pinti et al. 2019). Based on the relevant analysis of mitochondrial dysfunction in multiple organ tissues of T2D patients, it was found that the key factors leading to mitochondrial dysfunction were overproduction of ROS and decreased expression of antioxidant enzymes (Santos et al. 2001; Cho et al. 2013). Because mitochondria are the primary site of ROS production, mitochondrial DNA(mtDNA) and the lipids and proteins that make up the mitochondrial matrix are vulnerable to damage from high levels of ROS (Amaral et al. 2013). It has been reported that oxidative stress may lead to fragmentation of the mitochondrial network, decreased mitochondrial respiratory capacity, and damage to mitochondrial membrane integrity (Anello et al. 2005), suggesting that diabetic induced oxidative stress-related mitochondrial function impairment.

Related studies have found that T2D is also involved in mitochondrial epigenetic and epigenetic transcription regulation. It has become clear that a range of mtDNA mutations and single nucleotide polymorphisms are associated with T2D (Amaral et al. 2008). In the case of T2D, mtDNA will undergo methylation and/or hydroxymethylation. Methylation marks are observed on mtDNA-encoded mRNA (mt-mRNA) transcripts, and the enzymes found to promote this mRNAN1-methyladenosine (m1A) modification in mitochondria are TRMT10C and TRMT61B (Li et al. 2017; Safra et al. 2017) studies have found that m1A blocks typical A:U-base pairing suggests that m1A in mt-mRNA may interfere with the translation process within mitochondria, leading to abnormal protein synthesis within mitochondria, thereby impinging mitochondrial function (Li et al. 2017). Therefore, changes in the mitochondrial epigenetic transcriptome induced by T2D help to further explain mitochondrial dysfunction in this condition.

### Endoplasmic reticulum stress

The endoplasmic reticulum (ER), as a fine network of tubules, is involved in the cellular protein folding process (Schröder and Kaufman 2005; Hampton 2002). When the ER work load is too heavy to bear, the protein will not be able to reach the natural folding state, resulting in the long-term accumulation of unfolded or misfolded proteins in the ER lumen, which is called ER stress (Karna et al. 2020). Cells respond to ER stress through a tightly regulated and highly conserved signal transduction pathway (specific adaptation program) called the unfolded protein response (UPR) (Guzel et al. 2017) to maintain cell homeostasis and the ability of cells to adapt to adverse environments. Animal experimental studies have shown that oxidative stress and excessive production of ROS can change REDOX homeostasis in ER, thus disrupting ER protein folding and inducing ER stress (Malhotra and Kaufman 2007), and ER stress can in turn cause oxidative stress through oxidative protein folding (Cao and Kaufman 2014) and other pathways to produce ROS, which is manifested as the mutual promotion of the two. In streptozotocin induced diabetic rat model, it was found that diabetes can not only indirectly induce ER stress through the above oxidative stress pathway; It can also induce ER stress by inducing hyperglycemia, making AGEs, non-esterified fatty acids (NEFA) and other nutrients surplus, thereby directly inducing ER stress (Incalza et al. 2018).

## Potential mechanism of male infertility induced by diabetes

**Fig. 2:**
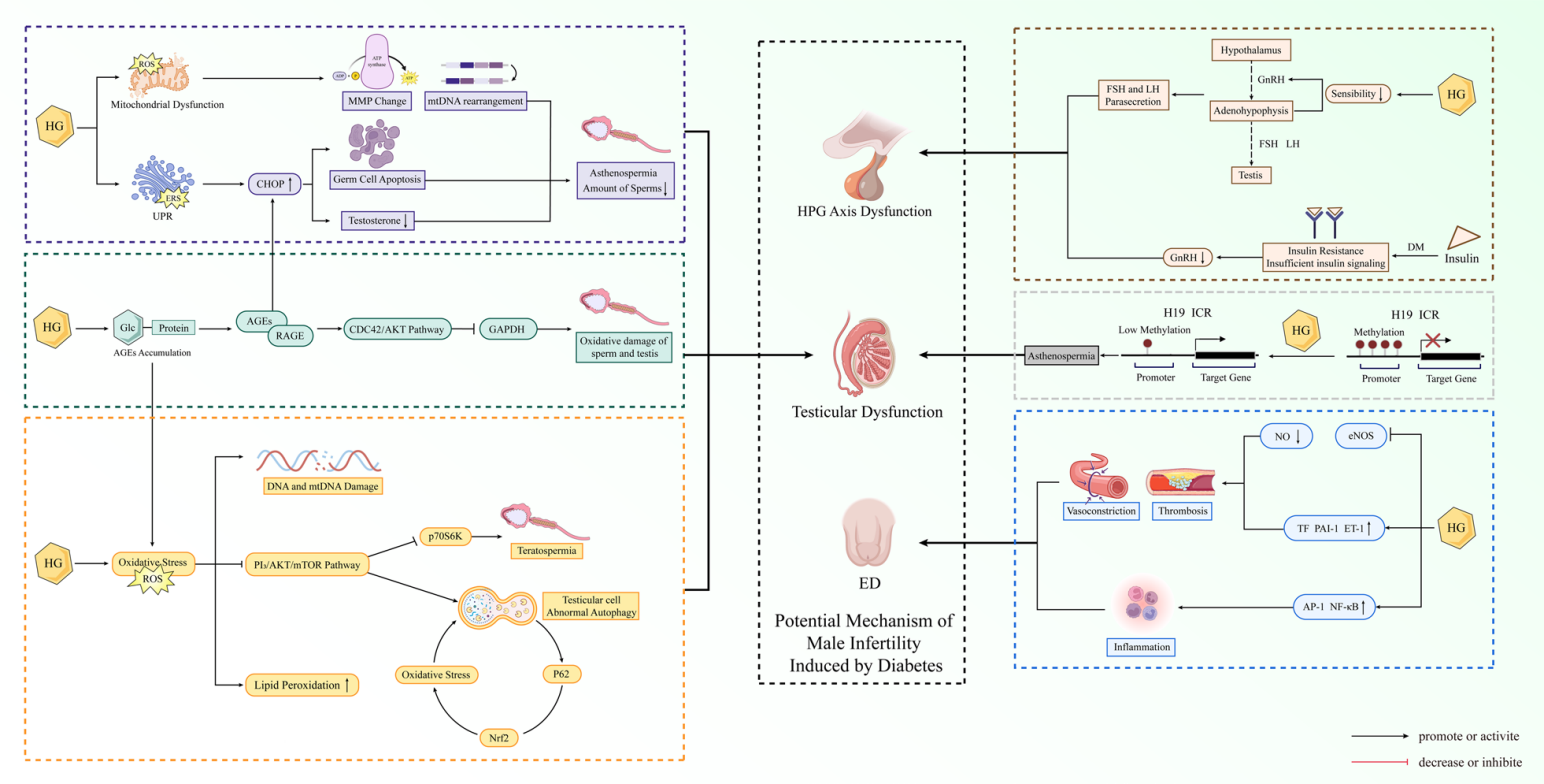
Potential mechanism of diabetes-induced male infertility. HG: high glucose; ROS: reactive oxygen species; UPR: unfolded protein response; MMP: mitochondrial membrane potential; CHOP: C/EBP homologous protein; AGEs: advanced glycation end products; RAGE: the receptor of advanced glycation endproducts; GAPDH: glyceraldehyde-3-phosphate dehydrogenase; PI_3_: phosphoinositide 3; mTOR: mammalian target of rapamycin; p70S6K: p70 ribosomal protein S6 kinase; Nrf2: Nuclear factor erythroid 2-related factor 2; ED: erectile dysfunction; HPG: hypothalamic-pituitary–gonadal axis; GnRH: Gonadotropin-Releasing Hormone; FSH: follicle-stimulating hormone; LH: luteinizing hormone; AP-1: activator protein 1; ET-1: endothelin-1; ICR: imprinting control area

The effects of diabetes on the reproductive system are multilayered and multifaceted, including HPG axis dysfunction, testicular dysfunction, ED (Fig. 2).

### Endocrine imbalance—dysfunction of HPG axis

The HPG axis is an important and classical Neuro-endocrine circuit in human body. The "transit station" of this pathway, the hypophysis secretes hormones including follicle-stimulating hormone (FSH) and luteinizing hormone (LH):FSH acts on sertoil cells of the testes to promote sperm maturation in fine seminiferous tubules; LH acts on Leydig cells of testis and promotes the increase of serum testosterone level (Page et al. 2008). A large number of studies have shown that the physiological changes that have adverse effects on the male reproductive endocrine system are mainly mediated by HPG axis. Through the negative feedback regulation of HPG axis, hyperglycemia exposure reduces the sensitivity of hypophysis to the stimulation of gonadotropin-releasing hormone (GnRH), resulting in abnormal secretion of LH and FSH, which affects the normal male reproductive function (Shi et al. 2017). In addition, the relevant review discussed the effect of insulin on the HPG axis, which is mediated by insulin receptors and associated signaling pathways (Schoeller et al. 2012a). The study found that the fertility ability of brain-specific insulin receptor knockout mice was significantly reduced, spermatogenesis was damaged, and interstitial cells atrophied. This suggests a link between insulin signaling in the brain and fertility (Schoeller et al. 2012a; Fujikawa et al. 2010). Further shows that insufficient insulin signaling in the brain of diabetic patients may reduce the output of hormones such as GnRH, which is necessary to preserve Leydig cell populations for successful stimulation of spermatogenesis (Brüning et al. 2000).

### Testicular dysfunction

Testicular dysfunction appears to be the most common underlying mechanism of male infertility. Testicular dysfunction can be divided into sperm-related damage and damage to the microenvironment surrounding the sperm. The former is mainly characterized by spermatogenesis disorder and sperm function impairment. The destruction of the microenvironment around sperm includes many aspects. For example, In vitro d-glucose culture experiments with mouse testicular fragments have shown that high D-glucose concentrations may lead to impaired function of supporting cells and inhibited proliferation, thus preventing effective regulation of spermatogenesis (Tavares et al. 2017). A comparison of the spatial maps that testis produced in normal and diabetic mice revealed the destruction of the spatial cells and tissues of spermatogenic tubules in diabetic mice, resulting in spermatogenic disorders (Chen et al. 2021). In addition, diabetes can also cause histological changes in testicular veins and the diameter of sertoil cells, resulting in decreased tissue perfusion and affecting testicular function (Mogaddami et al. 2018). The mechanism of testicular dysfunction is closely related to the above complex pathophysiological changes in diabetes.

#### Disorders of glucose metabolism affects sperm motility and fertilization

Studies have shown that normal glucose metabolism is an important event in spermatogenesis. Sperm depend on glucose for fuel to maintain basic cellular activity and specific functions such as motility and fertilization (Bucci et al. 2011). Sertoil cells also be needed to break down glucose to produce lactic acid to maintain spermatogenesis, and hormones such as FSH, testosterone, and insulin fundamentally control this process (Galardo et al. 2008). The transport of glucose between the blood and tissues is achieved by different membrane proteins, including active transport (sodium-dependent glucose transporter [SGLT]) or passive transport (glucose transporter [GLUT]) (Scheepers et al. 2004). In diabetic patients, GLUT consumption increases and expression decreases (Burant and Davidson 1994). The transport of glucose and other metabolic intermediates within testicular cells is highly controlled by the blood-testicular barrier mediated by GLUT so it is susceptible to influence in diabetic conditions (Bucci et al. 2011). GLUTs are also found in mature sperm (Bucci et al. 2011). In the case of diabetes, sperm glucose metabolism is impaired, resulting in low fertility and even infertility.

Hyperglycemia induced damage to male reproductive organs such as testis is mainly achieved through three biochemical pathways: advanced glycation end products (AGEs) formation pathway, diacylglycerol-protein kinase C (PKC) pathway and polyol path (Maresch et al. 2018). Based on a large number of studies, AGEs formation pathways are more common as injury mechanisms. Glycosylation is a common form of modification and is an initial necessary condition for the distribution of structural and functional components of sperm (Cheon and Kim 2015). In diabetic men, non-enzymatic accidental covalent binding between sugars and protein amino groups occurs under hyperglycemia conditions, resulting in excessive accumulation of AGEs (Singh et al. 2001). In the previous studies, The distribution of AGEs in the testis and epididymis of male heterozygote Ins2 Akita ± mice by immunohistochemistry and qRT-PCR showed that AGEs and Receptors for advanced glycation end products (RAGE) are widely distributed in the testis (Maresch et al. 2019). Studies have shown that AGEs can not only directly activate oxidative stress through the formation of crosslinks that alter protein structure and function (Karimi et al. 2011), but also activate downstream intracellular pathways of CDC42 and AKT1 through specific binding with RAGE, resulting in excessive superoxide production in mitochondrial electron transport chain, thus inhibiting the activity of glyceraldehyde-3-phosphate dehydrogenase (GAPDH). This eventually leads to oxidative damage to testis, sperm, etc. (Brownlee 2001).

#### Oxidative stress leads to oxidative damage of sperm

A large number of studies have shown that ROS increase and oxidative stress are the main causes of male infertility induced by diabetes. Low ROS levels play a positive regulatory role in sperm function. Both in spermatogenesis and during the movement of sperm from the testis to the encounter with the egg, low levels of ROS are important for improving sperm function, promoting sperm capacitation, and promoting sperm maturation and even fertilization (Ford 2004). However, human sperm is vulnerable to oxidative damage. When sperm mitochondria produce too much ROS and have exceeded the antioxidant capacity of sperm, it will aggravate the oxidative stress of sperm, resulting in oxidative damage (Aitken and Clarkson 1987).

The effects of oxidative stress on male reproductive organs such as testis and sperm can be manifested in the following aspects. The first is the direct effect of oxidative stress. The researchers believe that the fluidity of the cell membrane mainly depends on the lipid bilayer. High levels of ROS attack the polyunsaturated fatty acids in the sperm cell membrane, causing lipid peroxidation, which destroys the lipid bilayer on the cell membrane and disrupts the fluidity of the sperm cell membrane (Koppers et al. 2008) which can affect sperm function by affecting material transport, energy flow, immune defense, etc., leading to male infertility (Iwasaki and Gagnon 1992). Oxidative stress has been shown to be a major cause of damage to DNA integrity in the sperm nucleus and mitochondria (Agarwal et al. 1989). Damage to DNA integrity may accelerate sperm apoptosis, resulting in reduced sperm count and reduced semen quality (Agarwal et al. 2003). The DNA oxidation level in sperm of infertile male patients (Zini et al. 2009) and apoptosis level (Agarwal et al. 2003) in mature sperm were significantly higher than those in normal male patients, indicating that excessive ROS level would cause damage to sperm and accelerate the apoptosis of germ cells. By chemiluminescence assay, the early apoptotic marker of mature sperm in infertility patients was significantly increased compared with the mature sperm in the normal sperm donor control group (Agarwal et al. 2003).

Secondly, high blood glucose induced elevated ROS levels, can significantly inhibit the PI3/AKT signal pathway, then mTOR (p – mammalian target of rapamycin) signaling pathway is restrained, thus induced abnormal testicular cells autophagy (Lin et al. 2016). Unlike moderate autophagy, which plays a protective role in testicular injury caused by hyperglycemia (Sato et al. 2016), abnormal autophagy of testicular cells accelerates the degradation of autophagy related protein P62 in autophagy cells, and further inhibits the activation of Nuclear factor erythroid 2-related factor2 (Nrf2). Thus, the feedforward loop connecting P62 and Nrf2 is destroyed, the antioxidant capacity of testis is reduced, and the oxidative stress of testis is further aggravated, forming a vicious circulation (Tian et al. 2020). Autophagy causes extensive damage to the male reproductive system, such as inhibiting testicular Leydig cells, reducing serum testosterone levels (Zhao et al. 2018), inhibiting proliferation of supporting cells (Duan et al. 2016), and disrupting the integrity of the blood-testicular barrier (BTB) (Yi et al. 2018). In addition, inhibition of PI3/AKT/mTOR signaling pathway can inhibit the expression of p70S6K in sperm, resulting in sperm deformation and deformity, and can lead to reduced sperm parameters and motor activity, and even affect epididymal function (Xu et al. 2016). Other studies have shown that the inhibition of this pathway directly inhibits the proliferation of spermatogonia, affects the normal function of somatic cells and the reconstruction of BTB (Tian et al. 2020).

#### Mitochondrial dysfunction affects energy metabolism of germ cells

The influence of mitochondrial dysfunction on male fertility has been a hot topic in recent years. Mitochondria, as important energy organelles, are involved in the regulation of spermatogenesis and the proliferation, differentiation and apoptosis of germ cells in testis (Vertika et al. 2020). Mitochondria line the periphery of microtubules at the tail of sperm and produce large amounts of ATP, which provides energy for the movement of sperm flagella to maintain sperm motility (Vignera et al. 2015). When the mitochondrial function is damaged by diabetes, the ultrastructure and membrane potential of mitochondria are changed (Pelliccione et al. 2011), and energy production is limited, thus affecting spermatogenesis, motility and fertilization process. Recent studies have found that these injuries can be ameliorated by inositol (Condorelli et al. 2012). In addition, when mitochondrial dysfunction occurs, the intrinsic apoptotic pathway mediated by mitochondrial signaling is difficult to carry out and therefore fails to regulate apoptosis of primordial germ cells (PGC), which may lead to abnormal migration of PGC into the gonads, adversely affecting the male reproductive system (Bejarano et al. 2018). The long-range PCR and PCR—RFLP method detection of infertile men mutations in the mitochondrial genome, found 4977 and 7599 bp deletions of mtDNA are associated with spermatogenic failure (Talebi et al. 2018). Multiple mitochondrial DNA rearrangements characterized by decreased sperm motility are shown in male infertility (Kao et al. 1995). Although the correlation between mtDNA mutation and male infertility has been reported, the specific mechanism of the correlation needs to be further studied. Mitochondria are also involved in the metabolism of various sperm-related proteins. Based on a comparison of the proteome of diabetic and normal human sperm, significant differences were found in many proteins involved in spermatogenesis or fertilization (Agarwal et al. 2016). About half of these protein abnormalities are due to mitochondrial metabolic disorders. The protein interaction network shows that the reduction of cystatin C and dipeptidyl peptidase 4 in mitochondria may be an important reason for the decreased sperm motility in diabetic patients (An et al. 2018).

#### ER stress triggers the process of germ cell apoptosis

ER stress may be involved in the damage of male reproductive system, including testis, epididymis, sperm and other germ cells. Studies have suggested that ER stress may be a novel signaling pathway regulating male germ cell apoptosis, and male infertility may be the result of interference with ER stress during male reproduction (Karna et al. 2020). A study in male fruit flies reported that the ER stress gene is highly expressed in the male accessory gland and induces intense ER stress to reduce fertility (Chow et al. 2015). ER stress triggers an unfolded protein response (UPR), and in the case of excessive UPR activity exceeding threshold levels, cells deteriorate and ultimately trigger apoptosis (Williams et al. 2014). In metagenomic animals, UPR is activated by three protein sensors on the ER membrane: inositol requires kinase 1(IRE1), pancreatic ER Eukaryotic Translation Initiation Factor 2(eIF2α) kinase (PERK), and activation transcription factor 6(ATF6) (Liu et al. 2017). The binding of the ER luminal protein companion BiP/GRP78 to the UPR sensors prevents their signaling (Chow et al. 2015). Based on the UPR initiation competitive binding model, the unfolded/misfolded proteins in ER activate the three UPR sensors by competitively binding BiP/GRP78, thus initiating a downstream signal transduction path (Cao and Kaufman 2012). There is a common downstream molecular C/EBP homologous protein (CHOP) in the three signaling pathways, which can promote ROS production and enhance apoptosis. Other related molecules include c-JunN terminal kinase, caspase-12, etc. (Kim et al. 2013). In a study of torsion/post-torsion testicular injury in a rat model, apoptosis of germ cells in the testis was reported through upregulation of eIF2α and CHOP, which revealed the role of ER stress in testicular ischemia/reperfusion injury (Huang et al. 2012). In vitro studies have shown that AGEs formed during the progression of diabetes inhibit testosterone production by uprating GRP78 and CHOP in testicular interstitial cells (Zhao et al. 2016). These studies fully demonstrated the important role of CHOP in male reproductive dysfunction induced by ER stress. In addition, ER stress may also alter the cation channel (CatSper) of sperm, thus affecting sperm maturation and overactivation (Soni et al. 2016).

#### Chronic inflammation causes testicular endocrine disorders

Under the condition of diabetes, hyperglycemia is involved in the production of systemic inflammation. Chronic inflammation can cause testicular endocrine disorder and lead to male reproductive dysfunction by releasing inflammatory cytokines or directly and independently affecting the HPG axis and/or testicular cells (Jiang et al. 2022). For example, IL-1β and CCL2 inhibit androgen synthesis by inhibiting the activity of steroid-producing enzymes and ultimately induce apoptosis in Leydig cells (Jiang et al. 2020). TNF-α induces the production of molecules such as NO, which are necessary for the inflammatory response to be able to persist and can directly or indirectly affect spermatogenesis, damage the sperm membrane and reduce semen quality (Fraczek and Kurpisz 2007). The study found that the interaction between inflammation and diabetes further aggravates the impact of both on the male reproductive system. The related mechanisms mainly include excessive accumulation of ROS, excessive release of cortisol, decreased level of triiodothyronine, and misalignment of activin/statin/follistatin axis (Jiang et al. 2022). In addition, hyperglycemia induces inflammation of the male reproductive system, leading to sperm damage and male infertility, and may also be associated with the repolarization of tissue macrophages from the anti-inflammatory M2-like subtype to the pro-inflammatory M1-like subtype (Tavares et al. 2017).

#### Epigenetic change

Epigenetic modifications include DNA methylation, histone modification, chromatin remodeling, and post-translational modification of microrRNA, without involving changes in DNA sequence (Ding et al. 2015). Diabetes affects epigenetic modifications in spermatogenesis. It was found that proper DNA methylation is important for male germ cells. DNA methylation is catalyzed by three DNA methyltransferases (Dnmts), with Dnmt3a and Dnmt3b initiating de novo methylation to establish new methylation patterns (Okano et al. 1999). Dnmt1 is mainly involved in maintaining methylation pattern (Robert et al. 2003). Conditional mutations in Dnmt3a targeting the germ line can lead to spermatogenesis defects (Kaneda et al. 2004). H19 is an important imprinting gene in males of childbearing age and may serve as a biomarker for developmental defects in human sperm (Li et al. 2016a). 100% methylation of the H19 imprinting control area (ICR) is shown in normal individuals, while H19 hypomethylation is shown in diabetic infertile males (Poplinski et al. 2010). Furthermore, a significant positive correlation was observed between the degree of H19 hypomethylation and sperm count in oligospermia individuals, and sperm motility in asthenospermia (Dong et al. 2017). However, although abnormal methylation of SNRPN and MEST maternal-imprinted genes has been found in patients with oligospermia, the results of different studies have been disputed (Dong et al. 2017; Hammoud et al. 2010).

### Erectile dysfunction

Erectile dysfunction (ED) is a sexual dysfunction characterized by the inability to establish or maintain an erection sufficient for satisfactory sexual intercourse during sexual activity, which seriously affects men's self-esteem and almost all areas of life (including interpersonal relationships, family relationships, etc.) (Cui et al. 2018). Epidemiological studies have shown that the prevalence of ED in patients with diabetes is about 3.5 times higher than in patients without diabetes (Cui et al. 2018; Corona et al. 2014; Kouidrat et al. 2017). This indicates a high correlation between the two. Erection of the penis requires relaxation of the smooth muscle of the cavernous body.NO is the most important and effective vasodilator in this process (Leite et al. 2007). After the release of NO, it immediately spreads into the cavernous body and vascular smooth muscle cells, inducing the activation of soluble guanylate cyclase and the accumulation of cyclic guanosine phosphate (cGMP), promoting the relaxation of vascular and cavernous smooth muscle by reducing intracellular Ga2+, and increasing blood flow and intracavernous pressure, resulting in penile erection (Burnett and Musicki 2005). As mentioned above, hyperglycemia induces oxidative stress, which may lead to endothelial dysfunction, mainly manifested in: Endothelial nitric oxide synthase (eNOS) activity is inhibited, NO synthesis is reduced, thrombosis factors (such as tissue factor and plasminogen activator inhibitors1) or ET-1 production is increased, which induces thrombosis and vasoconstriction, and nuclear factor kappaB (NF-κB) and activator protein1 are increased, leading to tissue inflammation (Defeudis et al. 2015). All of these eventually lead together to ED. In addition, poor blood glucose control can also lead to lower serum testosterone levels and aggravate ED (El-Sakka et al. 2008).

However, diabetes leading to ED is not as simple a process as described above. The study found that the incidence of ED in diabetic patients increases with the number and severity of complications and comorbidities (Feldman et al. 1994). Complications of diabetes (e.g., cardiovascular disease, nephropathy, neuropathy) and comorbidities (e.g., hypogonadism, metabolic syndrome, and depression) all induce ED through the same or different mechanisms. Albuminuria is an independent risk factor for ED in patients with diabetic nephropathy (Chuang et al. 2012). At the same time, abnormal renal biosynthesis of l-arginine (a NO substrate) and increased production of asymmetric dimethylarginine (an endogenous inhibitor of NO synthase) can lead to decreased production of NO and ultimately ED (Baylis 2006).

## Potential therapeutic measures for diabetes-induced male infertility

### Drug therapy

#### Insulin

Insulin is mainly used in the treatment of T1D. It has been found that the effects of exogenous insulin on semen parameters of Akita diabetic mice with testicular atrophy are mainly manifested in the regeneration of spermatogenic tubules and testis, and the improvement of serum testosterone and LH levels to save damaged spermatogenesis and restore male fertility, and this series of effects are mainly mediated through the HPG axis (Schoeller et al. 2012b). In addition, the use of insulin can also improve autophagy disorder in epididymis tissue, interstitial hyperplasia and inflammation (Li et al. 2020).

#### Resveratrol (RES)

RES is a polyphenol compound that acts as a powerful antioxidant and has been shown to be effective in improving blood glucose levels in T1D rats as well as quantitative and qualitative parameters of sperm in diabetic conditions, such as sperm count and morphology, and mitochondrial sheath activity (Simas et al. 2017). In streptozotocin—and nicotinamide induced diabetic adult rats, resveratrol increased sperm count and motility and had a dose-dependent positive effect on DNA structural integrity compared to the untreated group (Bahmanzadeh et al. 2019).

#### Metformin

It is well known that metformin is widely used to treat T2D by increasing tissue sensitivity to insulin and glucose. At the same time, it is also involved in the blood glucose control of T1D (Kaul et al. 2015). In streptozotocin-induced diabetic mice, metformin has been shown to significantly improve sperm count, testicular proliferation, and testosterone levels, which may depend on elevated leptin levels and the expression of its receptor Ob-R in the testis of mice after administration of metformin (Annie et al. 2020). In addition, metformin improved the downregulation of genes associated with steroid production in diabetic male mice, resulting in increased serum and testicular testosterone levels, increased Leydig cell count, improved sperm parameters, and reduced sperm nuclear DNA fragmentation (Nna et al. 2019).

#### Pioglitazone

Low dose pioglitazone treatment can significantly reduce fasting blood glucose and malondialdehyde levels, improve tissue damage caused by diabetes, enhance antioxidant activity and reduce apoptotic activity, and have beneficial effects on spermatogenesis and steroidogenesis in adult diabetic rats (Hasan et al. 2020).

#### Chinese herb

*Dioscorea zingiberensis* (DZ) is a traditional Chinese herb that restores the integrity of BTB and mediates ZO-1 and Nrf2 to reduce oxidative stress damage (Zhou et al. 2020). *Gynura procumbens* (GP), while anti-inflammatory, is also anti- hyperglycemia and upregulates gene expression of proteins related to sperm maturation and sperm-egg interaction (Kamaruzaman et al. 2018).

#### Phosphodiesterase-5 inhibitors (PDE5is)

PDE5is is a first-line oral drug recommended by the World Health Organization (WHO) for the treatment of ED, and has also begun to be widely used for the treatment of diabetic ED, including sildenafil, tadalafil, Vardenafil, etc. (Vickers and Satyanarayana 2002). The mechanism of action of this class of drugs is to inhibit the expression of type 5 phosphodiesterase (PDE5) in the cavernous body, then increase the concentration of cGMP in vascular smooth muscle cells, then reduce intracellular Ga2+ and cause smooth muscle relaxation, then increase the blood flow of the cavernous body, and thus improve ED (Montorsi et al. 2004).

### Gene therapy

Gene therapy refers to the introduction of normal genes or other functional genes into the body by means of gene transfer to manipulate defective genes and alleviate diseases that do not respond to drug therapy (Alnasser 2021). A large number of studies have shown that gene therapy has been widely used in the research of cancer, genetic diseases, AIDS, cardiovascular diseases and other diseases, and most of them have successfully passed clinical trials, and began to be used as a new type of treatment for clinical patients. Gene therapy strategies related to male infertility patients have been conceived. Hsa-miR-100-3p provides a new epigenetic regulator for proliferation, DNA synthesis and apoptosis of human Sertoli cells by binding to SGK3, providing new clues for gene therapy of male infertility (Liu et al. 2021). For the gene therapy for male infertility caused by problems related to testicular and sperm gene expression, related studies have reported that through testicle-mediated gene transfer (TMGT) and sperm-mediated gene transfer (SMGT) to transfer genes from somatic cells into testicle and sperm, with sperm deliberately used as a gene transfer vector (Coward et al. 2007). Although these gene therapy strategies have not been directly demonstrated to be effective in treating diabetes-induced male infertility, they still serve as potential therapeutic measures. However, there are currently no studies that can guarantee the efficiency and safety of germline gene therapy (Tang and Xu 2020).

### Exercise improves testicular and sperm function

Studies have shown that exercise can prevent or improve the adverse effects of diabetes on male fertility. In male infertility caused by obesity and diabetes due to poor lifestyle, appropriate exercise may improve intra-testicular spermatogenesis and semen quality by increasing testicular antioxidant defense, improving apoptosis, reducing pro-inflammatory cytokine levels, and enhancing the steroid-producing process (Minas et al. 2022; Samadian et al. 2019). Different forms of exercise such as aerobic training or combined endurance and resistance training showed similar adjuvant therapeutic effects (Saremi et al. 2022; Parastesh et al. 2019). In addition, animal experiments have shown that diabetes often causes histological changes in the male reproductive system, especially changes in the diameter of testicular veins and sertoil cells and the thickness of convoluted seminiferous tubules, leading to tissue perfusion abnormalities of reproductive organs. Short and long-term regular exercise can improve the adverse changes in these histologies to restore normal tissue perfusion and effectively improve reproductive disorders (Mogaddami et al. 2018). A related systematic review and meta-analysis showed that combined aerobic and resistance training (CET) appears to be the best form of exercise for improving male fertility, but its overall efficacy remains controversial (Hajizadeh Maleki et al. 2022). In addition, considering the combination of moderate intensity exercise training and insulin therapy, it can effectively amplify the effect of insulin resistance associated diabetes induced intracellular apoptosis of testis (Samadian et al. 2019).

### Mesenchymal stem cell therapy

Mesenchymal stem cells (MSCs) are a class of cells with multidirectional differentiation potential and self-renewal ability (Fu et al. 2019). MSCs have the functions of immune regulation, anti-inflammatory, anti-apoptosis and improvement of oxidative stress (Kassis et al. 2008; Kim et al. 2015; Wang et al. 2017), and can secrete some important nutritional factors, such as hepatocyte growth factor (HGF),vascular endothelial growth factor (VEGF) and fibroblast growth factor (FGF) (Li et al. 2016b), so they are often used as a suitable choice for clinical treatment. Breast milk derived mesenchymal stem cells (Br-MSCs) are more reproducible and distinguishable than many types of stem cells of adult origin (Hassiotou et al. 2012). Studies have shown that Br-MSCs have a potential therapeutic effect on type 1 diabetes-induced male infertility (Khamis et al. 2020). Previous studies have shown that MSCs can migrate to the damaged site and have the ability to differentiate into local tissues of the damaged site under the chemotactic action of transforming growth faction-β1 (TGF-β1) (Zhang et al. 2016). In this study, the relative expression of human glyceraldehyde-3-phosphate dehydrogenase in rat testis also suggests that Br-MSCs can be nested in the testis of diabetic rats to repair varying degrees of diabetes-induced testicular damage (Khamis et al. 2020). By comparing untreated diabetic rats with those treated with Br-MSCs, it was found that in male diabetic rats with early intervention of Br-MSCs, the hypothalamic Kisspeptin-GnRH system, HPG axis and testicular steroid production were up-regulated, and testicular oxidative stress and lipid peroxidation (Francois et al. 2013) were also improved, which was shown as an increase in testicular antioxidant activity: The activities of GSH, SOD and CAT were significantly increased, while the activities of malondialdehyde (MDA) were decreased (Zhou et al. 2019). This series of changes resulted in an increase in the overall testicular weight and gonad index, while sperm count, activity and survival rate also recovered or even exceeded the normal physiological status (Hassan and Alam 2014). In addition, MSCs have anti-inflammatory and anti-apoptotic functions. Bone marrow mesenchymal stem cells can repair cadmium-induced testicular injury in rats by inhibiting mitochondrial apoptosis (Wang et al. 2017). The related mechanism may be the inhibition of NF-κBp65/TNF-α pathway, the mRNA expression of proapoptotic markers including Fas, FasL, Bax and Caspase-3 decreased significantly, and the mRNA expression of anti-apoptotic Bcl2 increased. This mechanism may also be applicable to MSCs against diabetes-induced apoptosis of testicular cells. Numerous studies have found that MSCs therapy may also be a new approach to treating ED in diabetics (Li et al. 2016b). At present, a large number of MSCs based clinical trials have been conducted worldwide, highlighting their therapeutic effects on diseases such as diabetes. Although MSCs therapy appears to be safe and well tolerated, donor heterogeneity, in vitro amplification, immunogenicity, and cryopreservation issues need to be addressed before MSCs are transferred from clinical trials to clinical treatment (Galderisi and Giordano 2014; Galipeau 2013).

### Microbial therapy

Animal studies have shown that AOS (alginate oligosaccharides) modified fecal microbiota transplantation (A10-FMT) improves the systemic and testicular microenvironment through the gut microbiota—testicular axis to improve spermatogenesis and semen quality in T1D patients (Hao et al. 2022). This method is a novel and promising therapeutic method, which is expected to be further studied. It has been suggested above that hyperglycemia induces inflammation of the male reproductive system, leading to sperm damage and male infertility, and may also be related to the repolarization of tissue macrophages from the anti-inflammatory M2-like subtype to the pro-inflammatory M1-like subtype. Related studies have confirmed that adeno-associated viruses that allow the exclusive expression of Jumonji domain-containing protein D3 (JMJD3) in macrophages can be generated under the macrophage-specific CD68 promoter in streptozotocin-induced diabetic male mice, thereby transducing testicular macrophages back to M2-like phenotype to exhibit an anti-inflammatory state to alleviate the negative effects of diabetes on sperm motility, serum and testicular testosterone levels (Zhu et al. 2022).

## Conclusion

This review briefly describes the complex complications of diabetes, the potential mechanisms of diabetes-induced male infertility, and common therapeutic measures. Although the association between diabetes and male infertility has been discussed, there is a lack of large cohort clinical studies and clinical data to prove this idea. And no independent indicators have been found to link diabetes with reduced male fertility.

In recent years, mesenchymal stem cell therapy and the regulation of intestinal microbiota—testicular axis have become the focus of research. Antidiabetic drug therapy may be effective in improving male reproductive problems by controlling blood glucose, such as fighting testicular oxidative damage, improving semen quality and steroid production. However, it is important to note that these drugs exhibit the exact opposite effect of the treatment at improper dosages (Tavares et al. 2018). It is reported that surgery may also be a potential treatment.

Although in the state of hyperglycemia, severe oxidative damage occurs in sperm. However, analysis of semen samples from men with T1D showed that compared with non-diabetic fertile men, the semen mRNA profile of these patients showed altered expression of 21 genes, many involved in stress response, DNA metabolism, and replication/repair (Mallidis et al. 2009). Some of these genes are up-regulated to act as sperm protective factors. SPATA20 is a novel testicle-specific protein that regulates transcription factors such as NF-κβ (Hayashi et al. 1993). NF-κβ is an important intracellular antioxidant (Nakamura et al. 1997). Due to increased oxidative damage of sperm DNA in diabetic men, the upregulation of SPATA20 may reflect an enhanced antioxidant response to oxidative stress in diabetic Settings. In addition, DNA replication and DNA damage nucleotide excision repair (NER) pathways may provide an alternative mechanism to base excision repair (the primary mode of repair) to remove oxidized DNA damage in the sperm genome (David et al. 2007). Therefore, targeting the upregulation of these sperm protective genes and enhancing DNA damage repair pathways appear to be new therapeutic approaches.

Although there are so many therapeutic measures, most of them are only limited to animal experiments, and more clinical trials or expanded test scope are needed to verify their effectiveness and safety before clinical application so as to provide patients with systematic clinical guidelines. In future studies, it is necessary to further explore the deeper cellular and molecular mechanisms of diabetes-induced male infertility, so as to find more therapeutic targets and achieve individualized and precise treatment of diabetic male infertility.

## Data Availability

Data are included within this article.
